# Prevalence and factors associated with restraints in mental health in-patient wards

**DOI:** 10.1192/bjb.2025.10164

**Published:** 2026-06

**Authors:** Sophia Senthil, Mithilesh Jha, Praveen Kumar

**Affiliations:** 1 Royal College of Psychiatrists, London, UK; 2 Nottinghamshire Healthcare NHS Foundation Trust, Nottingham, UK; 3 Lincolnshire Partnership NHS Foundation Trust, Lincoln, UK

**Keywords:** Prevalence, clinical, socioeconomic, restraints, in-patient treatment

## Abstract

**Aims and method:**

Restraints in mental health in-patient settings can negatively affect recovery. This study aimed to examine the prevalence and associated factors of restraint use. A retrospective cohort study was conducted in a rural NHS mental health trust in the UK, covering all adult in-patients from July 2020 to July 2021.

**Results:**

The prevalence of restraint was 34%. Factors associated with restraint included age 18–25 or ≥65 years, female gender, disability, long-term sickness benefits, detention under the Mental Health Act, frequent admissions and a diagnosis of depressive or severe mental illness. Statistically significant associations were found for age ≥65 years (odds ratio 3.920), Section 2 detention (odds ratio 5.72), more than ten previous admissions (odds ratio 5.672) and depressive disorders (odds ratio 3.478).

**Clinical implications:**

Restraint use remains common and is linked to identifiable risk factors. These findings support the need for targeted interventions to reduce restraint, particularly for high-risk patient groups.

The use of restraint in psychiatric in-patient settings remains controversial, raising significant ethical, legal and clinical concerns. Guidelines recommend that restraint should be used only as a last resort, with preference given to the least restrictive interventions.^1^ Despite a wide range of initiatives to reduce restraint over the past decade, ^2–8^ considerable variation in prevalence persists across in-patient services in the UK and internationally.^9^ National guidance, including the Department of Health’s Positive and Proactive Care (2014)^2^ and the Mental Health Act Code of Practice (2015)^10^, emphasises minimising restrictive interventions. NHS Benchmarking Network data^11^ reports prevalence of restraint across UK services, but refers specifically to adults with intellectual disabilities. There remains limited, up-to-date, nationally representative data for general adult mental health in-patients, restricting comparability across settings.

Earlier evidence, including a 2013 systematic review, identified several risk factors consistently associated with restraint: male gender, young adult age, minority ethnic background, schizophrenia, involuntary admission, aggressive or absconding behaviour, and the presence of male staff.^12^ More recent reviews have noted similar associations with young age, male gender, minority ethnic status, greater symptom severity and mood disorders. However, the overall evidence suggests that risk is multifactorial, and there remains a lack of high-quality, contemporary studies that can definitively establish predictive factors.^13^

## Method

We chose a retrospective cohort design for this quantitative study to investigate the clinical, demographic and social factors associated with restraint in in-patient wards.

Data were collected between 1 July 2020 and 31 July 2021, on all patients admitted to the Trust’s in-patient services, which included acute male and female wards, psychiatric intensive care, rehabilitation and older adult wards. Data were obtained for both patients who did and did not experience restraint during their admission. The data collection period preceded the implementation of the Mental Health Units (Use of Force) Act 2018^14^ in March 2023, reflecting the availability of routinely collected data and the timing of ethical approval.

Patient-related sociodemographic variables included gender, age, ethnicity, nationality, marital status, employment and receipt of disability benefits. Clinical variables included the number of admissions during the study period, previous admissions, legal status at the current admission, source of admission, ward admitted, primary diagnosis and length of stay. Service-related variables included the reason for restraint, type of restraint, timing of the restraint by staff shift and duration in minutes.

Restraint was defined according to the Trust’s policy, aligned with national guidance from National Institute for Health and Care Excellence^15^ and the Mental Health Act Code of Practice. This definition included any physical intervention intended to restrict movement.

Two routinely collected data-sets were used. The ‘admission data-set’ contained sociodemographic and clinical variables for all patients admitted between 1 July 2020 and 31 July 2021. Patients admitted multiple times during the period were counted once. The ‘restraint data-set’ contained similar variables for patients who experienced one or more restraint episodes during the same period, regardless of the number of admissions or restraint incidents.

The main outcome was whether a patient experienced at least one episode of restraint during the study period. Because patients could be admitted more than once and could experience multiple restraints, the data were not independent. To address this, unique identifiers (‘RiO number’ for patients and incident identifier for restraint events) were used to link and differentiate admissions and restraint incidents.

Data were collapsed by these identifiers and merged to produce a data-set that classified patients as restrained or not restrained during the study period. Pairwise deletion was used to handle missing values in individual patient records. Ethical approval was obtained from London – City and East Research Ethics Committee (Integrated Research Application System project ID 307838), the Health Research Authority and Health and Care Research Wales (ref. 22/PR/0368). Individual patient consent was deemed inappropriate owing to the secondary use of a large anonymised data set.

## Results

Of the 836 patients admitted, 53.6% (*n* = 448) were men and 46.4% (*n* = 388) were women. Patients aged ≥65 years comprised 22.7% of the sample, followed by those aged 36–45 years (17.7%). Most patients were White (87.2%). UK nationality was recorded for 69%, whereas nationality was not recorded for 26.4%. Marital status was recorded as single for 47.2% of patients, but was not available for 18%. In terms of employment, 33% received long-term sickness benefits, 21.7% were retired and status was unknown for 17.9%. Disability status was recorded for 19.4%. Accommodation status was unrecorded for 36% of patients; the most common recorded category was owner occupancy (16.9%).

The final analytic sample (*n* = 281), derived from merging restraint and admission data-sets, was divided into two groups: those who experienced restraint and those who did not during the study period (1 July 2020 – 31 July 2021).

Descriptive and logistic regression analyses were used to examine the association between sociodemographic and clinical variables and the use of restraint. Because the outcome was dichotomous (restrained versus not restrained), binary logistic regression was used to estimate univariate and adjusted odds ratios. Multivariate regression was performed with a backward selection model in SPSS version 21. Bivariate associations were assessed with chi-squared or *t*-tests, as appropriate.

Table 1 shows baseline sociodemographic and clinical characteristics of patients who were and were not restrained.

**Table 1:** Baseline sociodemographic and clinical characteristics

Variable studied	Categories	Not restrained		Restrained		*P*-value
		*n*	%	*n*	%	
Age, years	18–25	66	58.4	47	41.6	0.000
	26–35	131	72.0	51	28.0	
	36–45	113	76.4	35	23.6	
	46–55	80	84.2	15	15.8	
	56–65	80	72.1	31	27.9	
	≥65	120	64.2	67	35.8	
Gender	Female	268	69.1	120	30.9	0.375
	Male	322	71.9	126	28.1	
Ethnicity	Black and minority ethnic	24	70.6	10	29.4	0.945
	White/White British	547	70.0	234	30.0	
Nationality	Non-UK	35	74.5	12	25.5	0.451
	UK	400	69.2	178	30.8	
Marital status	Divorced/widowed	100	78.1	28	21.9	0.068
	Married/civil partner	109	67.3	53	32.7	
	Single	268	67.8	127	32.2	
Employment status	Employed	66	74.2	23	25.8	0.343
	Long-term sickness benefits	188	68.1	88	31.9	
	Another category	103	74.1	36	25.9	
	Retired	121	66.5	61	33.5	
Disability status	No	434	70.8	179	29.2	0.331
	Yes	109	66.9	54	33.1	
Mental Health Act on admission	Informal	269	83.5	53	16.5	0.000
	Section 2	236	61.0	151	39.0	
	Section 3	42	55.3	34	44.7	
	Not known or recorded	41	87.2	6	12.8	
Number of previous admissions	One	94	64.8	51	35.2	0.385
	Two	55	69.6	24	30.4	
	Three	43	69.4	19	30.6	
	Four	24	70.6	10	29.4	
	Five	22	73.3	8	26.7	
	Between six and ten	36	60.0	24	40.0	
	More than ten	20	52.6	18	47.4	
Admissions during 1 July 2020 and 31 July 2021	One	245	70.4	103	29.6	0.385
	Two	47	65.3	25	34.7	
	Three	18	66.7	9	33.3	
	Four	1	100.0	0	0.0	
	Five	1	25.0	3	75.0	
	Six	1	50.0	1	50.0	
Depressive disorders	No	406	69.8	176	30.2	0.008
	Yes	98	81.7	22	18.3	
Schizophrenic disorders	No	447	72.1	173	27.9	0.625
	Yes	57	69.5	25	30.5	
Severe mental illness	No	340	67.7	162	32.3	0.217
	Yes	145	72.5	55	27.5	
Tobacco misuse	No	299	69.1	134	30.9	0.98
	Yes	186	69.1	83	30.9	
Length of stay, days	Mean (s.d.)	66.77 (90.6)		95.2 (95.37)		*T* = −4.06

Among those who experienced restraint, the proportion of women (30.9%) was slightly higher than men (28.1%). Patients aged 18–25 years accounted for 41.6% of those restrained, followed by those aged ≥65 years (35.8%). Patients from minority ethnic groups comprised 29.4% of restrained patients, compared with 30% of White patients. UK nationality was recorded for 30.8% of those restrained, whereas nationality was unknown for 25.5%. Regarding marital status, 32.7% of restrained patients were married, 32.2% were single and 21.9% were divorced. A third (33.1%) of restrained patients had a recorded disability. In terms of employment, 33.5% were retired and 31.9% were in receipt of long-term sickness benefits.

Clinical variables showed that 44.7% of restrained patients were detained under Section 3 of the Mental Health Act, 39% under Section 2 and 16.5% were informal. Nearly half (47.4%) of restrained patients had more than ten previous admissions, 40% had six to ten admissions and 34.7% had two admissions during the study period. Recorded primary diagnoses among restrained patients included schizophrenia (30.5%), severe mental illness (27.5%) and depressive disorders (18.3%). Tobacco misuse was reported in 30.9%.

The mean length of stay for restrained patients was significantly longer than for non-restrained patients (95.2 *v.* 66.8 days). At the incident level, seated restraint accounted for 30.1%, standing for 23.6% and restrictive escort for 15.2%. Nearly half of incidents (46.5%) occurred during late staff shifts. The mean duration of restraint was 6.5 min (s.d. = 10.3) (Table 2).

**Table 2:** Analysis of restraint data

	Category	Frequency	%
Type of restraint	Physical restraint – kneeling	53	1.98
	Physical restraint – other (not listed)	106	3.96
	Physical restraint – prone	218	8.14
	Physical restraint – restrictive escort	406	15.15
	Physical restraint – seated	805	30.05
	Physical restraint – side	60	2.24
	Physical restraint – standing	632	23.59
	Physical restraint – supine	399	14.89
Timing when restrained, according to staff shift pattern	Early shift	809	30.20
	Late shift	1246	46.51
	Night shift	624	23.29
Duration of restraint, min	Mean ± s.d.	6.49	10.31

A binary logistic regression model was conducted to test the effect of sociodemographic and clinical predictors on restraint. Backward selection was used, with predictors entered simultaneously and removed step by step if their effects were not significant. This approach allowed assessment of the combined model at the outset, as well as how excluding variables affected remaining predictors.

Significant associations were observed for age ≥65 years, detention under the Mental Health Act, repeated admissions and depressive disorders (Table 3). Compared with patients aged ≥65 years, the probability of restraint was significantly lower in age groups 26–35 (odds ratio 0.090, 95% CI 0.019–0.432), 36–45 (odds ratio 0.068, 95% CI 0.015–0.303), 46–55 (odds ratio 0.092, 95% CI 0.015–0.572) and 56–65 (odds ratio 0.190, 95% CI 0.042–0.864) years. The youngest group (18–25 years) did not show a significant effect in the final model.

**Table 3:** Logistic regression model of factors associated with the use of restraints in mental health in-patient units

	*B*	s.e.	Wald statistic	Significance	Odds ratio	Exp(*B*) 95% CI	
						Lower	Upper
Age, years							
18–25	−0.466	0.768	0.368	0.544	0.628	0.139	2.827
26–35	−2.410	0.801	9.051	0.003	0.090**	0.019	0.432
36–45	−2.692	0.765	12.394	0.000	0.068***	0.015	0.303
46–55	−2.390	0.935	6.537	0.011	0.092*	0.015	0.572
56–65	−1.662	0.774	4.615	0.032	0.190*	0.042	0.864
≥65	1.844	0.978	3.487	0.079	5.920*	1.123	10.345
Name of ward admitted to							
Castle ward	−1.793	0.560	10.257	0.001	0.166**	0.056	0.499
Mental Health Act status							
Informal	−0.008	0.879	0.000	0.992	0.992	0.177	5.559
Section 2	1.744	0.887	3.865	0.049	5.720*	1.005	12.545
Section 3	1.755	0.966	3.299	0.069	5.781	0.870	38.389
Number of previous admissions							
One time	−2.907	0.778	13.974	0.000	0.055***	0.012	0.251
Two times	−1.594	0.787	4.104	0.043	0.203*	0.043	0.950
Three times	−0.652	0.734	0.788	0.375	0.521	0.124	2.197
Four times	−1.341	0.894	2.249	0.134	0.262	0.045	1.509
Five times	−1.759	0.932	3.562	0.059	0.172	0.028	1.070
Six to ten times	−0.883	0.745	1.406	0.236	0.413	0.096	1.780
More than ten times	1.811	0.988	3.286	0.069	5.672*	2.233	8.443
Diagnosis							
Depressive categories	1.246	0.575	4.693	0.030	3.478*	1.126	10.741

Omnibus *χ* ^2^(3) = 66.439, *P* < 0.001. The Cox and Snell *R* ^2^-statistic was 0.338 and the Nagelkerke *R* ^2^-statistic was 0.451, which indicated that the model was well fitted.

*P < 0.05, **P < 0.01,***P < 0.001.

Patients admitted to Castle Ward (an acute female ward) had lower odds of being restrained compared with other wards (odds ratio 0.166, 95% CI 0.056–0.499). Patients detained under Section 2 of the Mental Health Act had higher odds of restraint (odds ratio 5.720, 95% CI 1.005–12.545) compared with those admitted informally or under Section 3.

Compared with patients with more than ten previous admissions, those with one (odds ratio 0.055, 95% CI 0.012–0.251) or two previous admissions (odds ratio 0.203, 95% CI 0.043–0.950) were significantly less likely to be restrained. Patients with depressive disorders had significantly increased odds of restraint (odds ratio 3.478, 95% CI 1.126–10.741), whereas other diagnostic categories showed no significant effect.

## Discussion

The data collection period preceded the implementation of the Mental Health Units (Use of Force) Act 2018 in March 2023. This timing reflected the availability of routinely collected data and the date of ethical approval. We acknowledge that our findings may not fully capture practice changes introduced post-implementation.

The main finding was that 34% of admitted patients experienced at least one episode of restraint. In terms of social and demographic characteristics, patients who were restrained were more likely to be aged 18–25 or ≥65 years; be female; have a recorded disability; receive long-term sickness benefits or be retired; and have a marital status recorded as single, married or partnered. Clinically, restrained patients were more often detained under Mental Health Act Sections 2 or 3, had histories of frequent admissions, or had a recorded diagnosis of depressive disorder or severe mental illness compared with those who were not restrained.

Statistically significant associations were found for age, marital status, detention under the Mental Health Act, frequency of admissions and diagnosis. Sociodemographic and clinical variables are presented in Table 1, and regression results in Table 3.

Stepwise logistic regression confirmed independent associations with restraint for older age (≥65 years), detention under Section 2, more than ten previous admissions and depressive disorder. Among patients who were restrained, length of stay was also longer than among those who were not restrained.

Service-level factors such as staff gender composition and staffing ratios were not available in the routinely collected data-sets, and therefore were not analysed.

### Sociodemographic characteristics

The role of gender in restrictive interventions has been variably reported. Some studies have linked male gender to chemical restraint,^16,17^ whereas others suggest female patients are at higher risk.^18^ Studies specifically examining physical restraint often report no significant gender differences, implying comparable use across men and women.^19,20^ However, most of these studies are more than a decade old and largely involved involuntary populations, limiting generalisability.

Age has similarly been examined, with some studies reporting higher restraint rates among younger patients,^18^ others linking older age with greater risk, and some finding no relationship.^19,21^ In contrast, our study demonstrated a robust association between restraint and older age, particularly ≥65 years (odds ratio 3.920, 95% CI 1.123–10.345).

### Clinical characteristics

We found strong associations between restraint and detention under Section 2 of the Mental Health Act (odds ratio 5.72, 95% CI 1.005–12.545), more than ten previous admissions (odds ratio 5.672, 95% CI 2.233–8.443) and depressive disorders (odds ratio 3.478, 95% CI 1.126–10.741).

Previous studies have more often associated psychotic disorders with higher risk of restraint,^22–24^ although mood, organic (especially dementia), substance use and personality disorders have also been reported as risk factors.^25^ Severity of illness and specific symptoms such as hostility, suspiciousness and threats of violence are consistently linked to restraint, as is lower global functioning.^22,26,27^

In our study, depressive disorders were significantly associated with restraint. This may reflect presentations characterised by agitation, suicidality and difficulties with de-escalation during acute episodes. Staff perceptions of elevated risk in severe affective illness may also contribute to decisions to restrain.

Although multivariate analysis did not show ward-specific effects, univariate analysis suggested a possible increased risk on Castle Ward, which is an acute female ward.

Although some use of restraint is unavoidable when managing acute risk, our findings emphasise the importance of identifying high-risk groups for whom trauma-informed and preventative alternatives may reduce coercion. The results also suggest that restraint is not solely a response to patient behaviour, but reflects systemic influences such as patterns of detention and frequent admissions.

Models such as safe wards and trauma-informed care have demonstrated reductions in restraint through staff training, relational de-escalation and environmental adjustments. These approaches are particularly relevant for patients with recurrent admissions or depressive disorders, where early engagement and individualised care planning may prevent escalation.

Unlike many earlier studies that linked restraint mainly with schizophrenia and male gender, our findings highlight older adults and depressive disorders. This divergence reflects the evolving complexity of in-patient populations and suggests that assumptions about ‘typical’ high-risk groups may obscure opportunities for targeted intervention.

### Strengths and weaknesses of our study

Some major strengths of our study are the relatively large cohort size and inclusion of all admissions over a full year, providing robust prevalence estimates. Several sociodemographic and clinical factors were associated with restraint, notably older age (≥65 years), detention under Section 2, frequent admissions (>10) and depressive disorder.

Limitations must also be acknowledged. Missing data in key sociodemographic variables (e.g. marital status, nationality, accommodation) may have introduced bias. Pairwise deletion could have underestimated or overestimated associations. The retrospective design limited the capture of contextual factors, such as antecedents of restraint or available de-escalation strategies. Generalisability is restricted to similar rural UK settings. Only primary diagnoses were analysed; comorbidities and secondary diagnoses were not systematically recorded. Finally, the study was conducted prior to the implementation of Seni’s Law, which mandates enhanced monitoring and governance of restraint; current practice may therefore differ.

## Data Availability

The data that supports the findings of this study are available from the corresponding author, S.S., upon reasonable request.

## About the authors

**Dr Sophia Senthil**, MBBS, FRCPsych, is a consultant psychiatrist with Nottinghamshire Healthcare NHS Foundation Trust, Nottingham, UK; and an executive faculty member and co-academic secretary of the General Adult Faculty with the Royal College of Psychiatrists, London, UK. **Dr Mithilesh Jha**, MBBS, MRCPsych, is a consultant psychiatrist with Lincolnshire Partnership NHS Foundation Trust, Lincoln, UK. **Dr Praveen Kumar**, FRCPsych, is Inpatient Clinical Director and a consultant psychiatrist with Lincolnshire Partnership NHS Foundation Trust, Lincoln, UK.
